# SLA-Printed BaTiO_3_–Reinforced Bio-Nanocomposites: Influence of Printing Parameters on Mechanical, Dielectric, and Thermal Properties

**DOI:** 10.1021/acsomega.5c08292

**Published:** 2025-10-30

**Authors:** Tarlan Mahouti, Ece Örnek, Beste Çevir, Hale Berber, Mehmet Ali Belen, Hasan Sadıkoğlu, Hakan Yilmazer

**Affiliations:** † Department of Metallurgical and Materials Engineering, 52999 Yildiz Technical University, Istanbul 34220, Turkiye; ‡ Department of Chemical Engineering, Yildiz Technical University, Istanbul 34220, Turkiye; § Faculty of Engineering and Natural Sciences, Department of Electrical and Electronics Engineering, İskenderun Technical University, Hatay 31200, Turkiye; ∥ Health Biotechnology Joint Research and Application Center of Excellence, Istanbul 34220, Turkiye

## Abstract

The development of biocompatible nanocomposites with enhanced dielectric behavior has gained significant importance in medical electronics, particularly for high-frequency applications. This study presents the fabrication of barium titanate (BaTiO_3_)-reinforced nanocomposites using a photopolymer bioresin and stereolithography (SLA) 3D printing. Nanocomposites were prepared with 0.5, 1.0, and 1.5 wt % BaTiO_3_ concentrations and printed using a Creality HALOT R6 SLA printer. Postprocessing involved support removal, isopropyl alcohol cleaning, and UV curing for 120 s on both sides. Dielectric measurements were performed using a Vector Network Analyzer (VNA) in the 1–20 GHz range, where the dielectric constant of the pure resin was found to be approximately 3.06, but unexpectedly decreased to ∼2.58 at 1.0 wt % BaTiO_3_ (attributed to the low filler fraction limiting polarization). The loss tangent remained below 0.05 across the range, supporting low-loss characteristics. SEM-EDS analysis confirmed uniform filler dispersion without visible agglomeration. FTIR spectroscopy indicated the successful incorporation of BaTiO_3_ into the polymer matrix. Thermal analysis showed slightly enhanced stability, while mechanical testing revealed an increase in tensile strength from 25.3 MPa (neat resin) to 27.6 MPa for the 1.0 wt % nanocomposite. The combined results validate that BaTiO_3_-reinforced SLA-printed nanocomposites can meet the dielectric and mechanical performance requirements of GHz-range medical sensors without sacrificing print resolution or biocompatibility.

## Graphical abstract

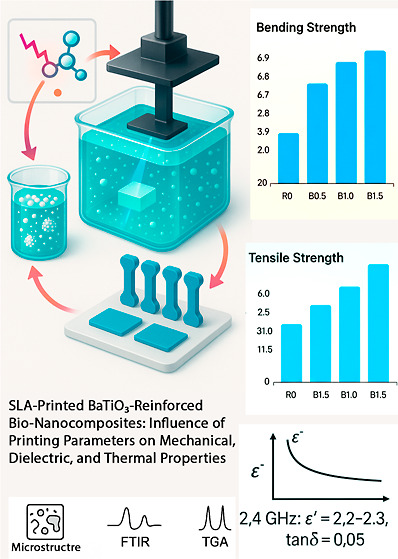

## 1 Introduction

The increasing integration of electronics into compact and high-performance systems has driven a growing demand for novel material platforms that combine miniaturization, dielectric functionality, and structural stability. In particular, applications operating in the gigahertz (GHz) frequency range—such as wireless communication units, sensing modules, and diagnostic tools—require materials with high dielectric constants and low loss characteristics.^1–5^ Although conventional polymeric materials offer excellent processability and biocompatibility, they often exhibit low relative permittivity (ε_r_) and insufficient electromagnetic response at high frequencies.^6–8^ As a result, there is a pressing need to develop polymer-based nanocomposites that not only maintain desirable mechanical and processing features but also exhibit enhanced dielectric performance suitable for GHz-range applications.

Barium titanate (BaTiO_3_), a ferroelectric ceramic with a perovskite-type crystal structure, has attracted significant attention as a dielectric filler due to its high relative permittivity, low dielectric loss, and excellent chemical and thermal stability. Its multifunctional properties—including piezoelectricity and pyroelectricity—further enhance its appeal for applications in high-frequency electronic systems.^9–12^ While BaTiO_3_ also exhibits promising biocompatibility, its primary role in nanocomposite formulations is to boost dielectric performance across GHz-frequency regimes. For example, BaTiO_3_ nanoparticles have shown negligible cytotoxicity in vitro, supporting their safety for biomedical use.^13^ However, effective incorporation of BaTiO_3_ nanoparticles into polymer matrices presents substantial challenges, including nanoparticle agglomeration, limited interfacial adhesion, and the resulting impact on viscosity, light penetration, and overall 3D printability.^14^ Recent studies confirm that tailoring polymer matrix architecture and filler–matrix interface can markedly improve composite dielectric, thermal, and mechanical properties.^15^ Copolymerization and interfacial adhesion effects are especially influential in determining high-frequency dielectric response.^16^ In parallel, biobased polymer dielectric composites continue to gain importance for energy and biomedical applications.^17^ These findings support the rationale of our SLA-based BaTiO_3_ nanocomposites and place our results in the context of the most recent literature.

Recent advancements in additive manufacturing—particularly stereolithography (SLA)—have enabled the precise fabrication of polymer-based nanocomposites with intricate geometries and tunable properties. SLA employs a layer-by-layer construction approach using UV-curable resins, allowing for exceptional resolution and dimensional accuracy.^18–22^ By adjusting resin formulations and fine-tuning printing parameters such as exposure time, light-off delay, and postcuring protocols, it is possible to control both the structural integrity and functional performance of the printed parts. This capability makes SLA a promising technique for producing high-frequency dielectric components and geometrically complex materials that would be difficult to achieve via conventional manufacturing methods.^23–28^

Despite the advantages of SLA, the integration of ceramic nanoparticles—such as barium titanate (BaTiO_3_)—into UV-curable resin systems presents notable challenges. The high refractive index of BaTiO_3_ can hinder UV light penetration, leading to nonuniform curing and reduced dimensional accuracy. In addition, the incorporation of such fillers increases the viscosity of the resin, which adversely affects flowability and layer uniformity during printing.^29–31^ These limitations demand precise optimization not only of the resin formulation but also of the processing conditions, including nanoparticle dispersion protocols, exposure times, layer thickness, and postcuring strategies.^32,33^ Addressing these factors is essential to ensure the successful fabrication of functional nanocomposites with reliable mechanical and dielectric performance. Unlike previous BaTiO_3_/polymer composite studies that typically required high ceramic loadings (often ≥10 wt %) to achieve large dielectric constant (ε′) gains,^15–17^ the present work demonstrates that GHz-frequency dielectric functionality can be realized with very low filler fractions (≤1.5 wt %). This is achieved through a two-stage SLA optimization strategy that maintains print fidelity and mechanical integrity. Furthermore, the dielectric results are quantitatively benchmarked against recent GHz-frequency literature, showing that our ε′ (∼2.2–2.3 at 2.4 GHz) and loss tangent (∼0.05) values compare favorably with or surpass many reported BaTiO_3_/polymer systems operating in the same frequency range.

In this context, the present study aims to develop and characterize a series of BaTiO_3_-reinforced photopolymer nanocomposites optimized for SLA-based additive manufacturing. A commercially available UV-curable bioresin was selected due to its low toxicity, biodegradability, and compatibility with 405 nm SLA printing systems. BaTiO_3_ nanoparticles were incorporated at concentrations of 0.5, 1.0, and 1.5 wt %, and uniformly dispersed using a multistage process involving magnetic stirring, mechanical blending, and ultrasonication. The resulting nanocomposites were printed using a Creality HALOT R6 SLA printer and postprocessed via isopropyl alcohol washing and dual-sided UV curing to ensure complete polymerization and structural integrity.

To evaluate the structural and functional performance of the developed nanocomposites, a comprehensive set of characterization techniques was employed. Morphological and elemental analyses were conducted using scanning electron microscopy (SEM) and energy dispersive X-ray spectroscopy (EDS), while Fourier transform infrared spectroscopy (FTIR) and thermal analyses (TGA/DTG)­provided insights into chemical structure and thermal stability. Mechanical properties were assessed through standardized tensile and flexural tests (ISO 527-2 and ISO 178), and dielectric performance was evaluated using a Vector Network Analyzer (VNA) over the 1–20 GHz frequency range, relevant for high-frequency dielectric and electronic applications.

This study aims to demonstrate that SLA-printed BaTiO_3_-reinforced nanocomposites can achieve a well-balanced combination of printability, mechanical robustness, and dielectric functionality. By optimizing nanoparticle loading and processing conditions, the proposed approach contributes to the development of advanced polymer-based materials suitable for GHz-frequency applications, including next-generation sensors, dielectric components, and high-frequency communication systems.

## 2 Materials and Methods

The stereolithography (SLA) printing experiments were carried out using a commercial UV-curable photopolymer resin (ANYCUBIC Bio-Resin) filled with barium titanate (BaTiO_3_) nanopowder,^10^ ^,^ ^12^ Detailed specifications of all raw materials are given in Section 2.1 Materials. BaTiO_3_ was incorporated into the resin at 0.5, 1.0, and 1.5 wt % to enhance dielectric performance in the GHz frequency range. The homogeneous resin–filler mixtures were prepared by mechanical stirring and ultrasonication, then printed with an SLA system operating at 405 nm. After printing, specimens were rinsed with isopropyl alcohol (IPA) to remove uncured resin and postcured under controlled UV exposure to ensure complete polymerization.

### 2.1 Materials

The matrix material ANYCUBIC Bio-Resin formulated for SLA systems with a 405 nm light source. Its composition includes polycaprolactone acrylate (30–60 wt %) for biodegradability, ethylene glycol diacrylate (25–40 wt %) as a cross-linker, and phenyl bis­(2,4,6-trimethylbenzoyl)-phosphine oxide (2–5 wt %) as a photoinitiator. BaTiO_3_ nanopowder with a tetragonal perovskite structure was chosen for its high dielectric constant and piezoelectric characteristics. IPA was used for postprint cleaning, and UV postcuring was performed in a controlled chamber.

### 2.2 Surface Modification of BaTiO_3_ Nanoparticles

To improve dispersion and interfacial compatibility with the polymer matrix of the resin, BaTiO_3_ nanoparticles were subjected to three modification strategies

- **HNO**_**3**_ hydroxylation: 1 g BaTiO_3_ was dispersed in 2% HNO_3_ solution, stirred for 90 min, centrifuged (7000 rpm, 15 min), washed four times with deionized water, dried overnight at 80 °C, and heat-treated at 105 °C for 1 h.
- HCl hydroxylation: A similar protocol was followed using 5% HCl solution.
- Thermal annealing: BaTiO_3_ powders were annealed at 900 °C to modify surface crystallinity.

Despite improved surface hydroxylation, dielectric characterization revealed no significant enhancement from surface-modified BaTiO_3_ compared with untreated powders. Therefore, unmodified BaTiO_3_ was used in all final nanocomposite formulations.

### 2.3 Preparation of Nanocomposite Resins

BaTiO_3_ was incorporated into the resin at 0.5, 1.0, and 1.5 wt % using a standardized three-step protocol: (i) magnetic stirring for 30 min to wet the nanoparticles, (ii) mechanical blending at 1500 rpm for 5 min, and (iii) probe ultrasonication for 15 min in an ice bath to ensure deagglomeration. The suspensions were used immediately after preparation, and no sedimentation was observed prior to printing.

### 2.4 SLA Printing

Printing was carried out using an SLA system (CREALITY HALOT R6). For each batch, 200 mL of resin was poured into the vat. Digital models were sliced with manufacturer-recommended software, and parameters were optimized for structural fidelity: 50 μm layer thickness, initial exposure 25–35 s, subsequent layer exposure 3–5 s, motor speed 2 mm/s, 5 mm lifting height, and 4 s light-off delay. Three bottom layers were applied to improve build-plate adhesion. Each run lasted approximately 2 h, producing ∼45 g of material per batch. Printed parts were detached with a flat spatula, rinsed in isopropyl alcohol (IPA), and manually cleared of support structures. Final UV postcuring was performed per side to ensure complete polymerization and enhance mechanical stability.

### 2.5 Experimental Design and Sample Coding

#### 2.5.1 Stage 1–Parameter Optimization (R0–R7)

Pure resin specimens were fabricated under varying SLA conditions (layer thickness 0.025/0.05 mm, light-off delay 0/4 s, bottom layers 3/4) and postprocessing protocols (UV curing only, Soxhlet extraction, or UV curing + Soxhlet extraction) in Table 1. Soxhlet extraction involved 4 h IPA washing followed by drying at 80 °C overnight. Mechanical and dielectric results identified R0 as the optimal parameter set.

**Table 1:** Experimental Matrix of SLA Parameters and Post-processing Methods (all Samples Printed With Motor Speed = 2 mm/s and Rising Height = 5 mm)

code	layer thickness (mm)	light-off Delay (s)	exposure time (s)	bottom layer count	post-processing method	description
**R0**	0.05	0	25	3	UV curing only	wiped with isopropyl alcohol, UV cured (60 s per side, λ = 365 nm)
**R1**	0.025	0	35	4	UV curing only	same UV protocol applied with adjusted SLA settings
**R2**	0.05	0	25	3	Shower + Drying (Soxhlet only)	washed in Soxhlet with IPA for 4 h, dried overnight at 80 °C
**R3**	0.025	0	35	4	Shower + Drying (Soxhlet only)	alternative SLA setup; processed similarly to R2
**R4**	0.05	4	25	4	Shower + Drying (Soxhlet only)	modified exposure delay to assess photopolymerization efficiency
**R5**	0.05	0	25	3	UV curing + Shower + Drying (Soxhlet)	first UV cured (60 s per side), then Soxhlet (4 h) and oven-dried overnight at 80 °C
**R6**	0.025	0	35	4	UV curing + Shower + Drying (Soxhlet)	lower thickness; post-UV treated and Soxhlet extracted
**R7**	0.05	4	25	4	UV curing + Shower + Drying (Soxhlet)	with light-off delay; full postprocessing sequence applied

#### 2.5.2 Stage 2–Nanocomposite Fabrication (B0.5–B1.5)

Using R0 parameters, nanocomposite resins containing 0.5–1.5 wt % BaTiO_3_ were prepared (Figure 1) via the same dispersion protocol and printed under identical conditions (Table 2).

**Figure 1:**
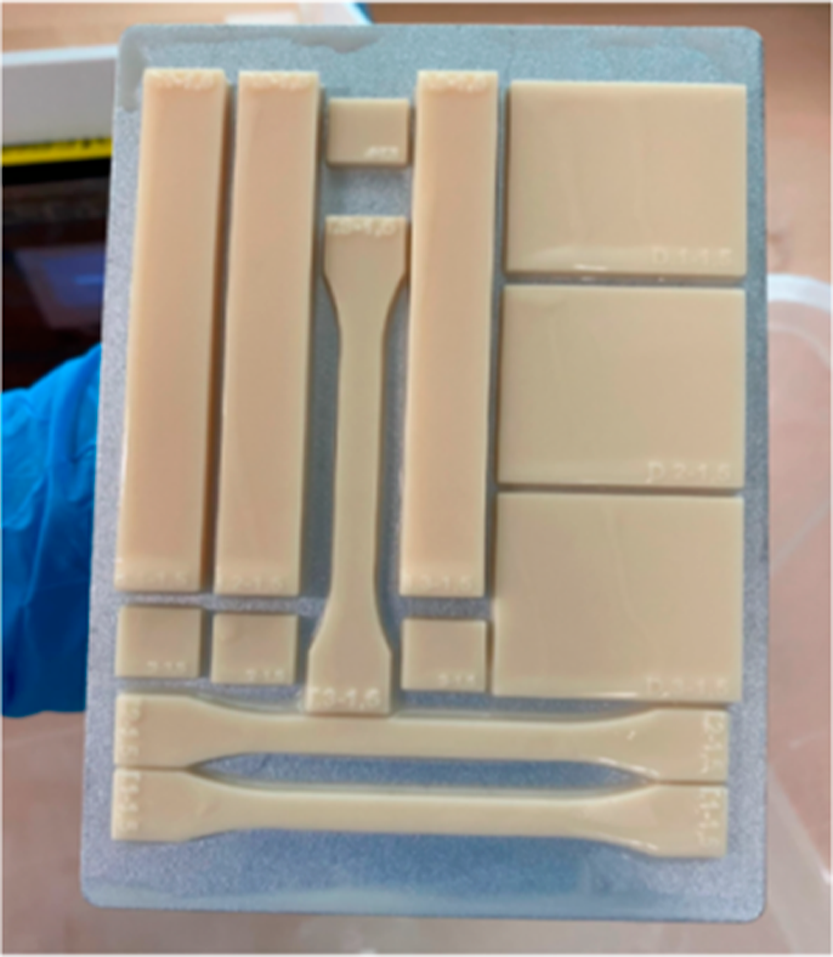
Samples containing 1.5% BaTiO_3_ on the printing platform.

**Table 2:** BaTiO_3_–Reinforced Nanocomposite Sample Set (Stage 2)

Samples	BaTiO_3_ Content (wt %)	SLA Parameters	Description
**R0**	0	Optimized	Neat resin (from Stage 1)
**B0.5**	0.5	Same as R0	Resin with 0.5 wt % BaTiO_3_
**B1.0**	1.0	Same as R0	Resin with 1.0 wt % BaTiO_3_
**B1.5**	1.5	Same as R0	Resin with 1.5 wt % BaTiO_3_

## 3 Characterization Techniques

To comprehensively assess the influence of BaTiO_3_ incorporation on the structural integrity, mechanical performance, chemical composition, thermal stability, and dielectric behavior of the developed photopolymer nanocomposites, a multimodal characterization strategy was implemented. The following techniques were employed in a systematic manner to ensure reproducibility and in-depth analysis across all sample groups.

### 3.1 Tensile Test

The tensile behavior of the samples was evaluated in accordance with ISO 527-2:2012 standards. All tests were carried out at room temperature using a universal testing machine, with a crosshead speed of 5 mm/min. Specimens were fabricated in Type 1BA geometry to ensure consistency with standardized protocols. A minimum of three replicates were tested for each sample group to ensure statistical significance.

### 3.2 Three-Point Bending Test

The bending/flexural properties of the printed samples were evaluated in accordance with ISO 178 using the same universal testing machine employed in tensile testing. The support span length was set to 64 mm, and the test was conducted at a crosshead speed of 2 mm/min. Rectangular beam-shaped specimens were utilized for all bending tests. For each composition, a minimum of three replicates were measured to ensure reproducibility.

### 3.3 Chemical Characterization

Fourier-Transform Infrared (FTIR) spectroscopy was performed using a PerkinElmer Frontier spectrometer equipped with an attenuated total reflectance (ATR) accessory. Spectra were collected in the range of 4000–400 cm^–1^ at a resolution of 4 cm^–1^, with 32 scans averaged per sample. No additional sample preparation was required.

### 3.4 Morphological Analysis

The surface morphology and nanoparticle dispersion characteristics of the printed nanocomposites were investigated using a ZEISS EVO LS10 scanning electron microscope (SEM) operated at an accelerating voltage of 10 kV. Prior to imaging, all samples were sputter-coated with a thin layer of gold to enhance surface conductivity and image resolution.

To assess the elemental composition and spatial distribution of barium and titanium, Energy Dispersive X-ray Spectroscopy (EDS) analysis and elemental mapping were performed.

### 3.5 Thermal Characterization-Thermogravimetric Analysis (TGA/DTG)

The thermal stability and decomposition behavior of the nanocomposites were assessed using a PerkinElmer TGA 4000 instrument. Approximately 5–10 mg of each sample was heated from 30 to 600 °C at a constant heating rate of 10 °C/min under a nitrogen atmosphere (flow rate: 20 mL/min) to prevent oxidative degradation.

### 3.6 Dielectric Characterization

The dielectric properties, including the real part of the permittivity (ε′) and the loss tangent (tan δ), were evaluated using a Vector Network Analyzer (VNA) over a broadband frequency range of 1–20 GHz. The measurements were performed at ambient temperature.

Nanocomposite samples were fabricated in cuboidal geometry and mounted into a coaxial dielectric test fixture designed for high-frequency applications. Prior to data acquisition, calibration was carried out using open, short, and load standards to ensure measurement accuracy and repeatability. The results offered critical insight into the suitability of the BaTiO_3_-reinforced resins for GHz-range medical and telecommunication applications.

## 4 Results and Discussion

### 4.1 Effect of SLA Printing Parameters on Pure Bio-Resin

To assess the influence of stereolithography (SLA) printing parameters on material performance, eight distinct groups of neat bioresin samples (coded R0–R7) were fabricated using varied combinations of layer thickness, exposure time, light-off delay, and postprocessing strategies (e.g., UV curing vs Soxhlet extraction). This initial analysis provides a foundation for identifying the optimal printing configuration prior to the incorporation of ceramic fillers, and serves to decouple the influence of printing artifacts from intrinsic material performance.

### 4.2 Dielectric Performance

The dielectric constant (ε′) of all pure bioresin samples exhibited a decreasing trend with increasing frequency, a typical behavior attributed to the declining influence of dipolar polarization at higher frequencies. Among the tested groups, sample R0 (corresponding to D303U) consistently showed the highest dielectric performance across the full frequency spectrum, with a maximum ε′ value of 3.06 at 2 GHz, gradually decreasing to 2.03 at 20 GHz. This indicates a superior energy storage capability, particularly in the lower GHz range. The loss tangent (tan δ), representing dielectric dissipation, showed a frequency-dependent increase in all samples. R0 again outperformed other groups by maintaining a relatively low tan δ of 0.07 at 1 GHz. However, this value increased to 0.72 at 20 GHz, reflecting higher energy losses at elevated frequencies. In contrast, R6 (D305US) exhibited the lowest dielectric constant and among the lowest loss tangent values, indicating reduced dielectric responsiveness but potentially favorable for low-loss applications in selective frequency bands.

Notably, samples subjected to Soxhlet-assisted postprocessing or surface treatments—such as R6 and R7—demonstrated modest reductions in ε′ but improved stability in tan δ at mid to high frequencies. This suggests that such processing routes may suppress residual monomer content or enhance polymer network uniformity, thereby reducing dielectric losses.

### 4.3 Mechanical Properties

The mechanical behavior of the bioresin samples was systematically assessed through uniaxial tensile and three-point bending tests (Figure 2). Among all tested formulations, sample R0 exhibited the highest tensile strength, averaging 22.65 MPa, which underscores its superior resistance under tensile loading conditions. In contrast, other groups showed markedly lower tensile performance, with values ranging from 10.20 MPa (R7) to 11.29 MPa (R2). These differences are likely attributable to variations in postprocessing protocols and resulting polymer network cross-linking density.

**Figure 2:**
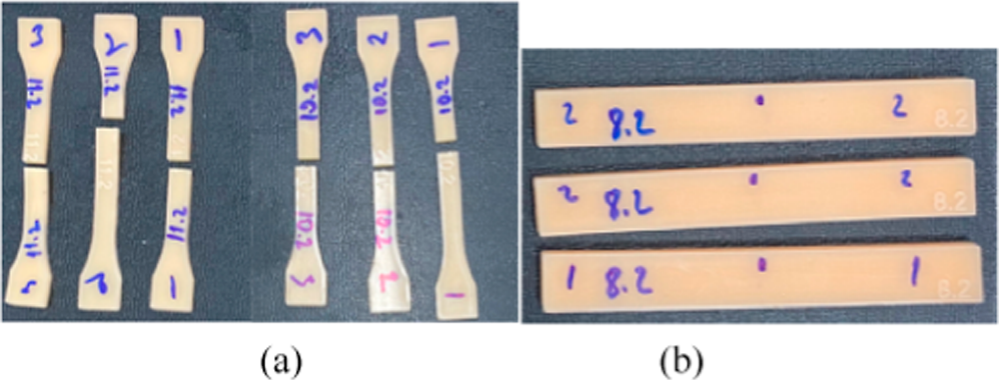
Representative images of the printed samples after (a) tensile and (b) 3-point bending tests.

Flexural performance followed a similar trend. Sample R0 again demonstrated the highest flexural strength of 26.33 MPa, outperforming R2 and R1, which yielded 18.93 and 16.28 MPa, respectively. These findings indicate that R0 offers a balanced combination of stiffness and toughness, potentially resulting from optimized layer adhesion and postcuring conditions.

### 4.4 Overall Comparison and Material Suitability

A comparative analysis of the mechanical and dielectric test results (Supporting Information, Table S1) underscores the superior performance of sample R0, which consistently demonstrated the highest tensile strength, flexural strength, and dielectric constant across the evaluated frequency spectrum. This highlights its suitability for high-performance applications requiring both structural robustness and reliable electromagnetic behavior—particularly within the low-to-mid GHz range (1–10 GHz), which is relevant for biomedical telemetry and IoT applications.

While samples R2 through R7 offered marginal improvements in select parameters—such as slightly lower loss tangents in certain frequencies or smoother surface finishes after postprocessing—they consistently underperformed in terms of mechanical integrity or dielectric efficiency (Figures 3 and 4). This indicates a clear performance trade-off: ultrasonic postprocessing and aggressive cleaning (e.g., Soxhlet extraction) may enhance surface or chemical characteristics but compromise structural cohesion and electromagnetic responsiveness.

**Figure 3:**
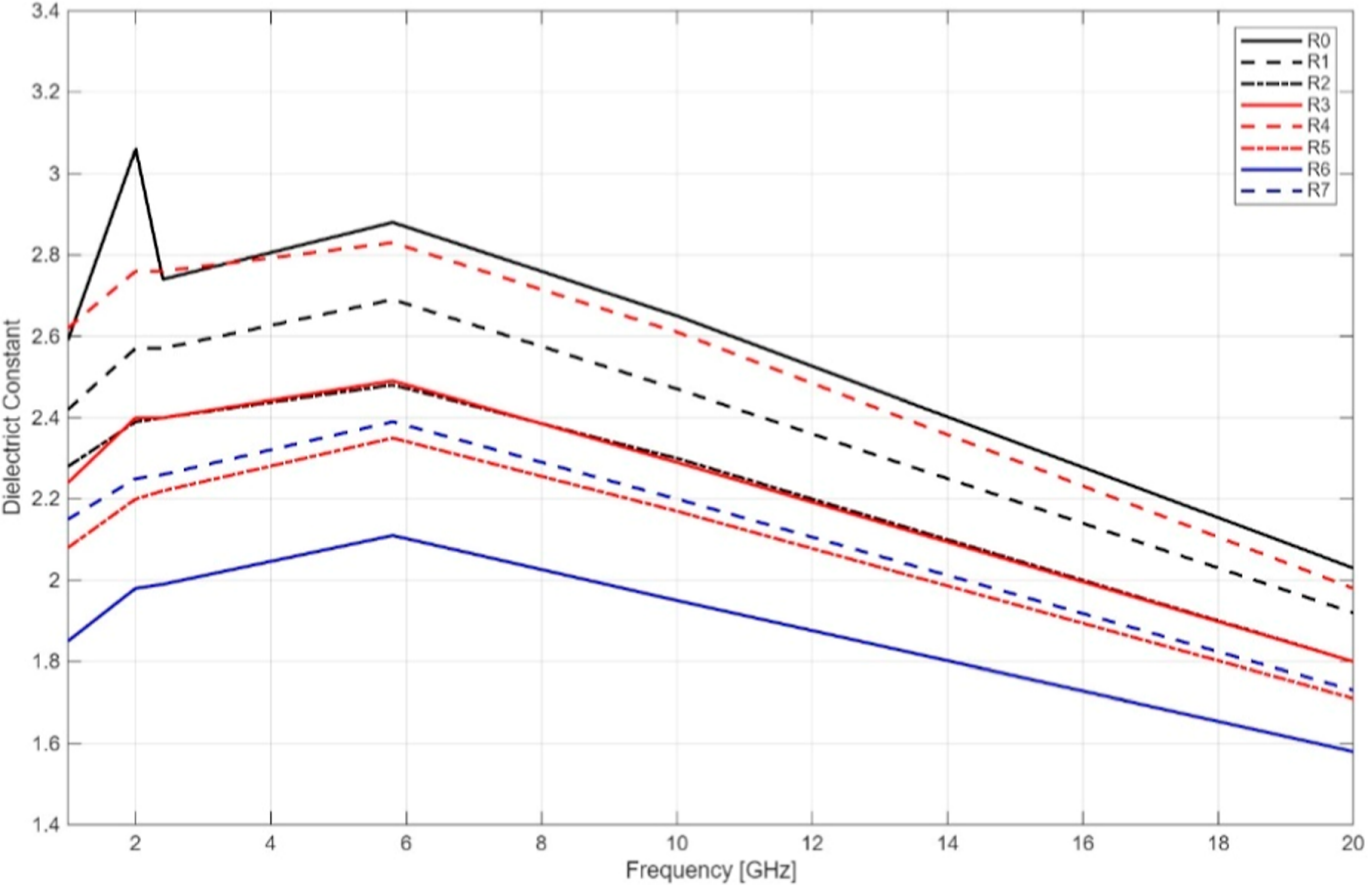
Dielectric Constant of the printed resin samples without BaTiO_3_ contents.

**Figure 4:**
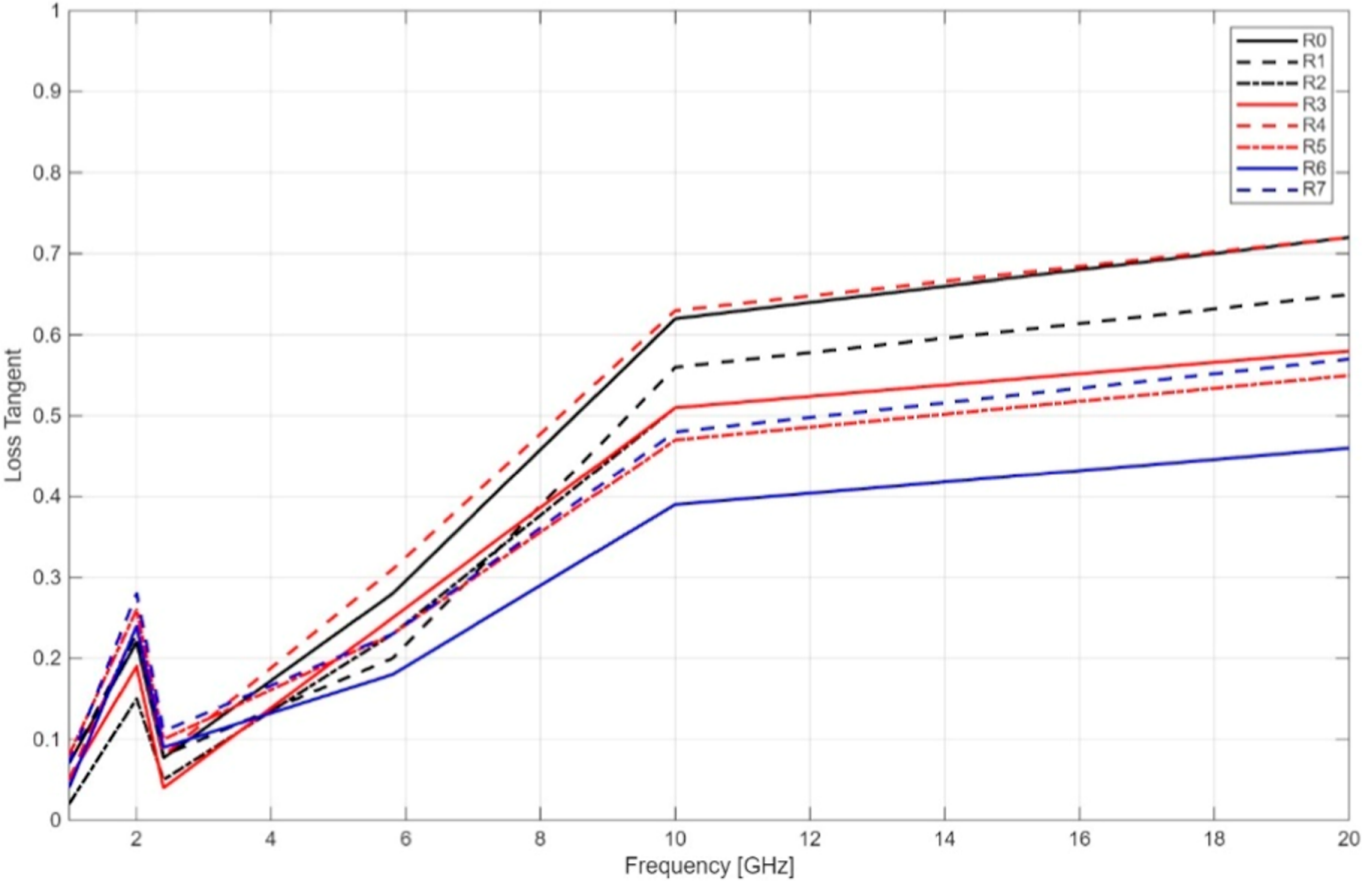
Loss Tangent of the printed resin samples without BaTiO_3_ contents.

In specific applications where low dielectric loss is prioritized over mechanical strength—such as electromagnetic shielding or passive sensing platforms—formulations like R6 or R7 may still offer utility. However, for applications requiring integrated mechanical and dielectric functionality, R0 emerges as the most balanced and reliable formulation.

### 4.5 Optimization Outcome

Following a comprehensive evaluation of mechanical and dielectric characteristics, the R0 configuration emerged as the optimal stereolithography (SLA) printing setup. This formulation consistently exhibited the highest tensile and flexural strengths as well as superior dielectric performance across the 1–20 GHz spectrum. Its balanced performance indicates a well-polymerized structure with minimal internal defects, effective UV curing, and stable electromagnetic behavior—critical factors for functional nanocomposite fabrication.

The optimized SLA parameters for R0 were as follows.

- Layer thickness: 50 μm
- Light-off delay: 4 s
- Exposure time per layer: 25 s
- Bottom layer count: 3
- Rising height: 5 mm

This configuration was adopted as the baseline printing condition for all subsequent experiments involving BaTiO_3_ reinforcement. The consistency and reproducibility offered by the R0 settings allowed for the isolation of nanoparticle effects without introducing additional variability from the printing process.

### 4.6 Effect of BaTiO_3_ Addition on the Optimized Bio-Resin

Building upon the optimized SLA printing parameters identified in sample R0, a series of BaTiO_3_-reinforced nanocomposites were fabricated to investigate the effect of high-dielectric ceramic fillers on the structural and functional performance of the bioresin. Barium titanate (BaTiO_3_) nanoparticles were incorporated at concentrations of 0.5 wt % (B0.5), 1.0 wt % (B1.0), and 1.5 wt % (B1.5) using a standardized dispersion protocol to ensure homogeneous distribution within the photopolymer matrix.

The resulting nanocomposites were subjected to mechanical testing, dielectric spectroscopy, and microstructural evaluation (SEM/EDS) to elucidate the correlation between filler content and performance. This stage aimed to determine the optimal ceramic loading that would enhance dielectric behavior without compromising the mechanical integrity or printability of the SLA-fabricated structures.

Figure 5 presents FE-SEM micrographs of 3D-printed nanocomposite surfaces fabricated with increasing concentrations of barium titanate (BaTiO_3_). Each subimage includes magnified regions to illustrate nanoparticle distribution more clearly.

**Figure 5:**
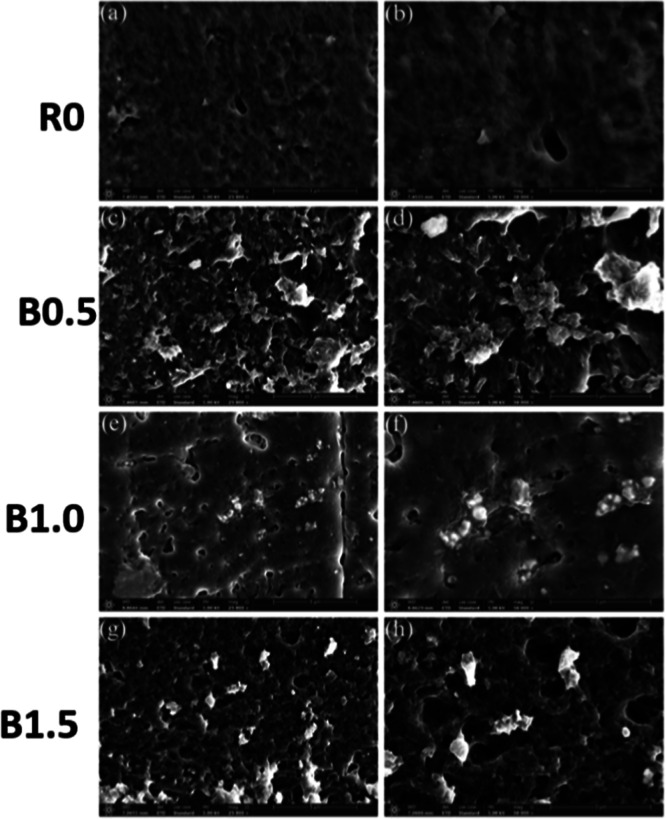
FE-SEM images of the printed samples with different BaTiO_3_ contents: (a,b) surface morphology of pure resin sample (R0); (c,d) surface morphology of 0.5 wt % BaTiO_3_-reinforced sample (B0.5); (e,f) surface morphology of 1.0 wt % BaTiO_3_-reinforced sample (B1.0); (g,h) surface morphology of 1.5 wt % BaTiO_3_-reinforced sample (B1.5).

In the reference sample (R0; Figure 5a,b), the surface appears smooth and homogeneous, confirming the absence of particulate fillers and the uniformity of the pure bioresin. Upon incorporation of 0.5 wt % BaTiO_3_ (B0.5; Figure 5c,d), the surface morphology becomes slightly more textured. Although individual nanoparticles are not easily distinguishable due to the low filler content, initial signs of localized agglomeration and nonuniform dispersion are visible. At 1.0 wt % loading (B1.0; Figure 5e,f), BaTiO_3_ nanoparticles are distinctly observed, exhibiting more uniform dispersion and better interfacial integration with the resin matrix. This level of reinforcement appears to enhance both resin-filler interaction and structural homogeneity, aligning with the improved dielectric and mechanical properties previously reported. In contrast, the 1.5 wt % sample (B1.5; Figure 5g,h) demonstrates increased surface roughness and reduced dispersion quality. The micrographs indicate the formation of larger agglomerates and possible sedimentation, suggesting that the percolation threshold for effective dispersion has been exceeded. Consequently, particle–particle interactions dominate over resin-particle interactions, leading to diminished morphological uniformity. These findings suggest that 1.0 wt % BaTiO_3_ represents the optimal loading for achieving effective nanoparticle dispersion, resin compatibility, and performance balance in SLA-printed nanocomposites.

Table 3 presents the Energy Dispersive X-ray Spectroscopy (EDS) spectra of SLA-printed nanocomposite samples fabricated with increasing concentrations of BaTiO_3_. As anticipated, the elemental contents of barium (Ba) and titanium (Ti) varied proportionally with the filler content. In the neat bioresin (Table 3), the EDS spectrum shows only carbon and oxygen—56.5 and 43.5 wt %, respectively—consistent with an inorganic-filler-free organic matrix. Upon introducing 0.5 wt % BaTiO_3_, barium and titanium become detectable at 0.3 and 0.1 wt %, while the matrix remains C/O-dominated; converting these elemental signals to a surface-equivalent BaTiO_3_ content using the stoichiometric mass fractions of BaTiO_3_ (Ba 58.89 wt %, Ti 20.53 wt %) yields ∼0.51 wt % (Ba-based) and ∼0.49 wt % (Ti-based), in excellent agreement with the nominal loading within the quantification uncertainty (±0.1 wt %). For B1.0, Ba and Ti increase to 1.0 and 0.6 wt %, corresponding to ∼1.70 wt % (Ba-based) and ∼2.92 wt % (Ti-based) BaTiO_3_ at the analyzed surface. The Ba-based estimate—less sensitive to spectral overlap for these low levels—indicates a surface concentration moderately above the bulk nominal value, which may reflect surface enrichment of BaTiO_3_ during curing (e.g., modest migration/settling effects) given the density contrast between the ceramic and the resin; nevertheless, this interpretation should be viewed as a plausible tendency rather than a definitive mechanism because EDS probes only the near-surface. At 1.5 wt % overall loading (B1.5) Ba and Ti are 0.7 and 0.3 wt %, giving ∼1.19 wt % (Ba-based) and ∼1.46 wt % (Ti-based), slightly below the target and suggestive of a more distributed (less surface-rich) particle arrangement at higher filler levels. Nitrogen is additionally observed in B0.5 (∼5.5 wt %) and B1.5 (∼8.8 wt %), which is consistent with nitrogen-bearing constituents in the resin/additive package or minor surface contamination; its presence does not affect the above stoichiometric conversions for BaTiO_3_.

**Table 3:** Shows EDS Spectra of the Printed Samples With Different BaTiO_3_ contents^a^

	R0		B0.5					B1.0					B1.5				
element	C	O	Ti	Ba	C	N	O	Ti	Ba	C	N	O	Ti	Ba	C	N	O
atomic (at.) %	63.4 ± 0.3	36.6 ± 0.2	0.0 ± 0.0	0.0 ± 0.0	57.2 ± 0.3	5.4 ± 0.5	37.3 ± 0.2	0.2 ± 0.0	0.1 ± 0.0	57.9 ± 0.3	6.7 ± 0.5	35.1 ± 0.2	0.1 ± 0.0	0.1 ± 0.0	59.9 ± 0.3	8.5 ± 0.5	31.5 ± 0.2
weight (wt) %	56.5 ± 0.2	43.5 ± 0.3	0.1 ± 0.0	0.3 ± 0.1	50.4 ± 0.2	5.5 ± 0.5	43.8 ± 0.3	0.6 ± 0.0	1.0 ± 0.1	50.6 ± 0.2	6.8 ± 0.5	40.9 ± 0.3	0.3 ± 0.0	0.7 ± 0.1	53.2 ± 0.2	8.8 ± 0.5	37.2 ± 0.2
net counts	467.8	85.4	652	922	593.1	6.4	121.3	2.7	3.1	525.7	6.9	94.7	1.2	1.7	480.8	7.2	67.7

a The ± deviation values under values of at % and wt % are the error values of the SEM-EDS detector for each element.

Figure 6 also includes EDS elemental distribution maps for Ba and Ti. Overall, the EDS results confirm successful incorporation of BaTiO_3_ and reveal a modest, loading-dependent variation in its near-surface representation relative to the bulk nominal content. These maps confirm a trend toward increasing clustering and declining uniformity at higher filler concentrations, particularly in sample B1.5 (Figure 6e,f), consistent with the morphological findings discussed in Section 4.6.

**Figure 6:**
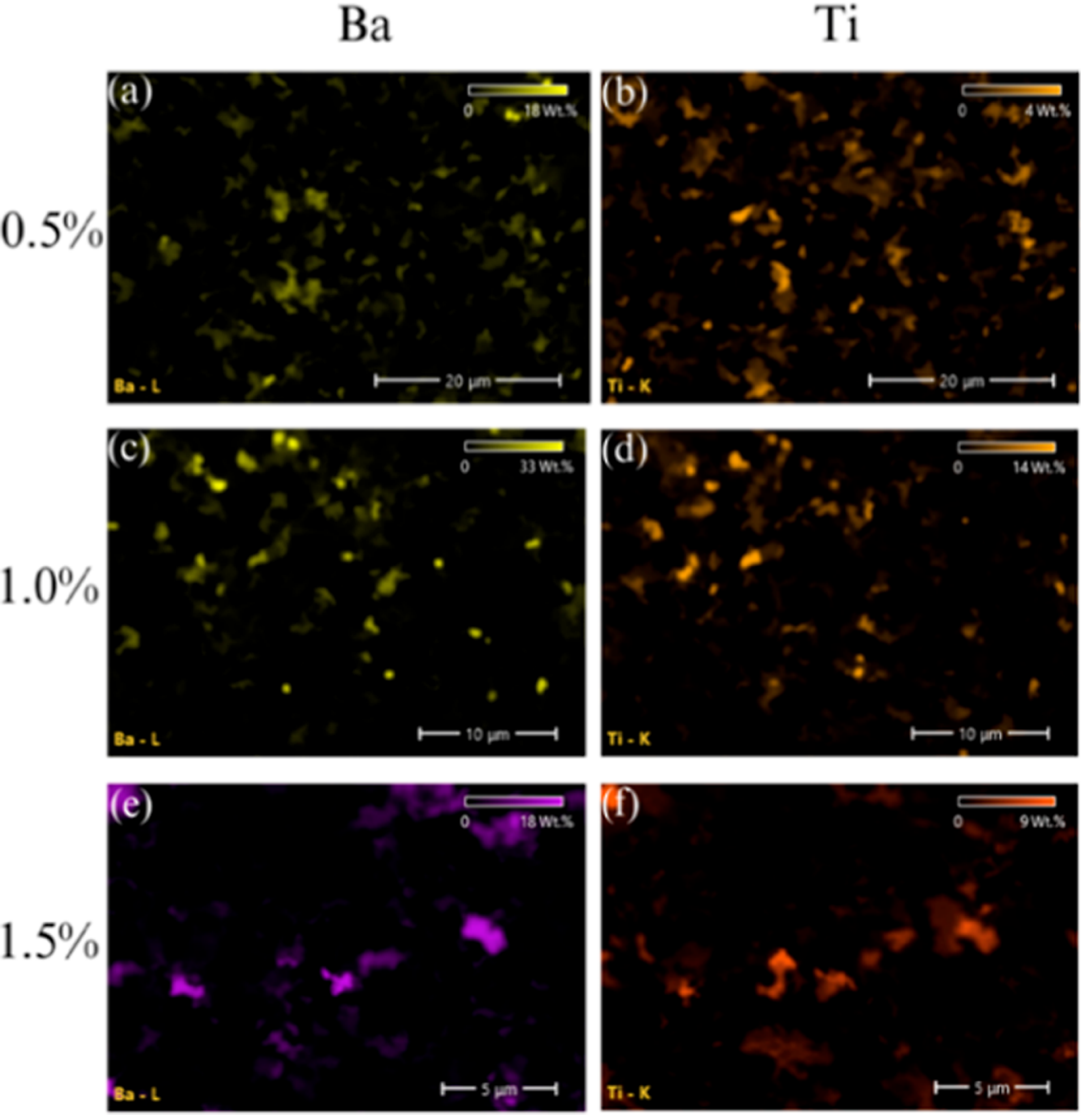
Element distribution map obtained from EDS analysis of the printed samples with different BaTiO_3_ contents: (a,b) Ba and Ti elemental mappings of the 0.5 wt % BaTiO_3_ sample, (c,d) Ba and Ti elemental mappings of the 1.0 wt % BaTiO_3_ sample, and (e,f) Ba and Ti elemental mappings of the 1.5 wt % BaTiO_3_ sample.

### 4.7 TGA Analysis

Thermogravimetric (TG) and derivative thermogravimetry (DTG) analyses were carried out to examine the thermal behavior of the neat SLA bioresin (R0) and the BaTiO_3_-reinforced nanocomposites containing 0.5 wt % (B0.5), 1.0 wt % (B1.0) and 1.5 wt % (B1.5) of ceramic filler. The corresponding TGA and DTG curves are presented in Figure 7, and the quantitative degradation data are summarized in Table 4. T_5_, T_10_ and T_50_ designate the temperatures at which the material has lost 5%, 10% and 50% of its initial weight, respectively. *T*_max_ denotes the temperature of maximum mass-loss rate (DTG peak) Together with the final residue measured at 800 °C, these parameters describe when decomposition begins, how rapidly it progresses, and how much solid matter survives high-temperature treatment.^34^ ^,^ ^35^

**Figure 7:**
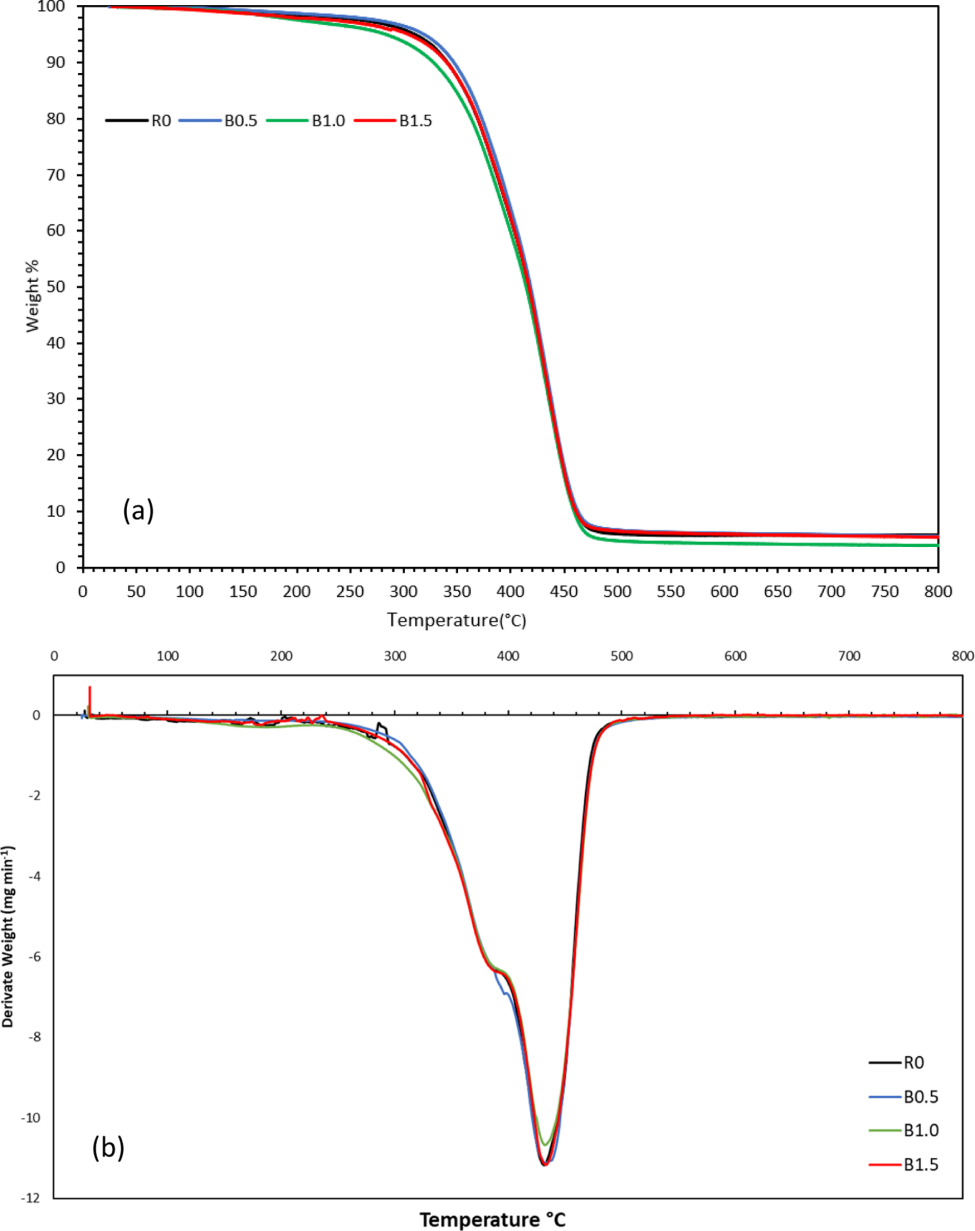
Thermogravimetric analysis (a,b) DTG curves of the printed samples with different BaTiO_3_ contents.

**Table 4:** Thermal Degradation Data of the Printed Samples With Different BaTiO_3_ Contents

sample	T_5_ (°C)	T_10_ (°C)	T_50_ (°C)	*T*_max_ (°C)	temperature range of degradation (°C)	residue at 800 °C (%)
R0	304.59	340.30	417.10	431.76	216–550	5.39
B0.5	319.04	346.95	418.70	433.46	248–556	5.53
B1.0	283.55	327.88	414.50	432.30	246–530	3.93
B1.5	310.30	340.94	417.31	433.03	233–548	5.80

The neat SLA resin (R0) exhibited a characteristic one-step degradation profile typical of photopolymerized acrylate networks. Its thermogravimetric (TG) curve showed only a minor mass loss of about 2% below ∼200 °C, attributable to the release of absorbed moisture and trace volatiles.^34^ The main decomposition of R0 then occurred sharply between approximately 250 and 500 °C, with the maximum rate of weight loss (DTG peak) at T max ≈432 °C as the organic polymer backbone undergoes scission. Beyond ∼550 °C, little further mass change was observed and around 5.4% residue remained at 800 °C, representing charred carbonaceous remnants of the resin.^35^ The DTG curve of R0 is relatively narrow and centered, indicating a predominantly single-stage decomposition with only a faint shoulder, consistent with a uniform network breakdown mechanism.

With 0.5 wt % BaTiO_3_ loading (B0.5), relative to R0, the T_5_ and T_10_ temperatures increased by 14.5 and 6.7 °C, respectively and the *T*_max_ shifted slightly higher temperatures (433.46 °C), with a small rise in residue (≈0.14 wt %). This points to mild stabilization consistent with ceramic fillers acting as local heat sinks and diffusion barriers and with limited chain-mobility near well-dispersed particles.^34^ ^,^ ^36^ On the other hand, by increasing the BaTiO_3_ loading to 1.0 wt % (B1.0), the temperatures of T_5_, T_10_ and T_50_ dropped below those of the pure sample while *T*_max_ increased only by 0.5 °C. The residue was the lowest (≈3.93 wt %). Such early scission is typical of particle–particle interactions and agglomeration, which diminish barrier effectiveness and may introduce microvoids/defects or weak interfaces that accelerate volatilization,^36^.^37^

At the highest BaTiO_3_ content studied (1.5 wt %, sample B1.5), a modest increase in *T*_max_ by 1.3 °C and the highest residual amount (≈5.80 wt %) were detected compared to sample B1.0. The T_5_ value increased to 310.30 °C, while the T_10_ and T_50_ values were almost the same as those of the neat sample, R0. However, the thermal degradation data obtained for this sample did not reach the values obtained for sample B0.5. This suggests a partial re-establishment of a filler network (improving heat-sink and barrier effects), albeit with remaining heterogeneity compared to the well-dispersed low-loading case.^35^ ^,^ ^37^

Consequently, for BaTiO_3_ reinforced bionanocomposite systems, at low loading, well-dispersed BaTiO_3_ can (i) absorb/redistribute heat (heat-sink),^38^ (ii) hinder gas diffusion (barrier effect),^38^ and (iii) locally restrict chain mobility via polymer–filler interactions—collectively delaying degradation. At intermediate loading, agglomeration/poor dispersion reduces the effective interfacial area, creates defects, and offsets the stabilization.^39^ At higher loading, partial network formation can recover some stability, though not necessarily beyond the optimum low-loading case.^40^ Moreover, the thermal degradation trends observed by TGA/DTG were consistent with the SEM micrographs, where the enhanced dispersion at 1.0 wt % BaTiO_3_ correlates with increased the degradation temperatures and improved the thermal stability, while the observed agglomeration and sedimentation at 1.5 wt % BaTiO_3_ coincide with an earlier degradation and accelerated mass loss.

### 4.8 Fourier Transform Infrared Spectroscopy (FT-IR)

The FT-IR spectroscopy was conducted to evaluate the chemical structure of the printed nanocomposite samples containing varying BaTiO_3_ ratios and to reveal the interactions between polymer matrix and nanoparticles. Figure 8 presents the FTIR spectra of pure and BaTiO_3_-reinforced resin samples.

**Figure 8:**
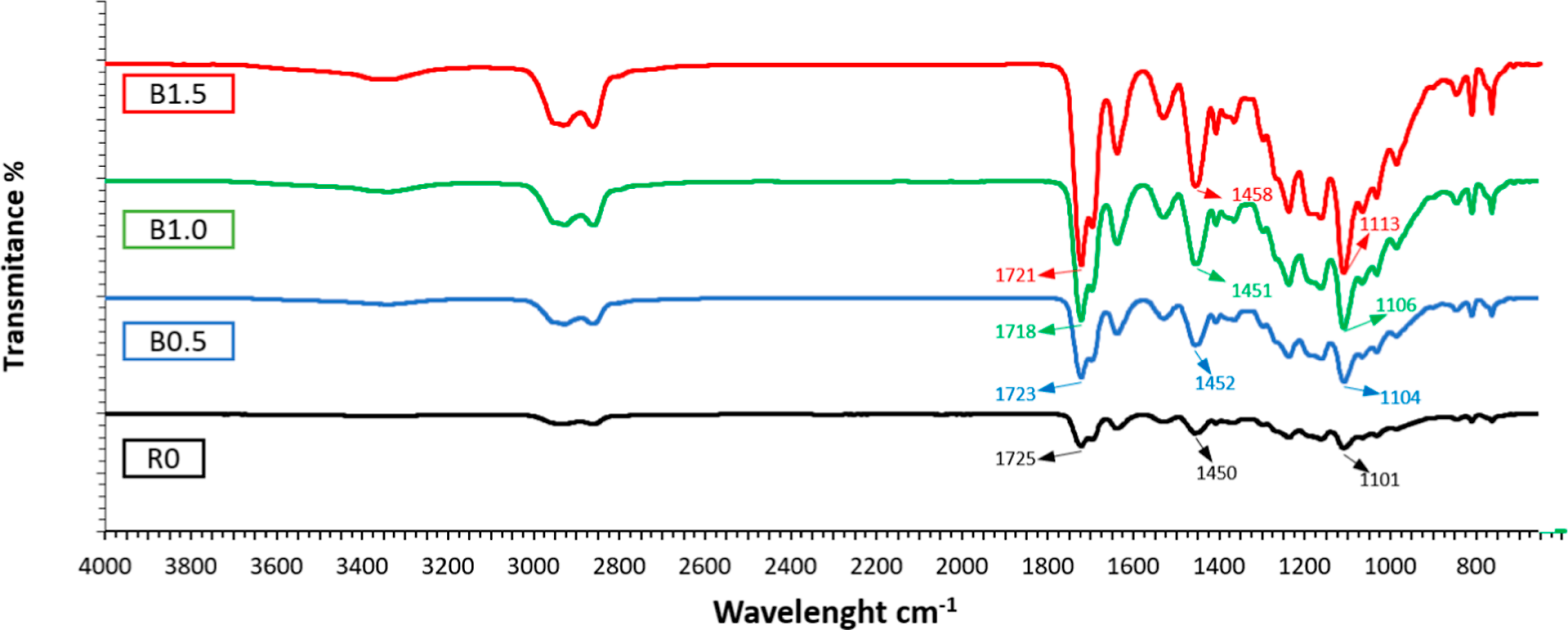
FTIR spectra of the printed samples with different BaTiO_3_ contents.

For the neat resin, a prominent absorption peak was observed in the range of ∼1720–1730 cm^–1^, corresponding to the C=O stretching vibration of ester bonds, which reflects the characteristic backbone structure of the polymer matrix composed of EGDA (ethylene glycol diacrylate) and PCLA (polycaprolactone acrylate).^41^ Additionally, peaks in the 1100–1300 cm^–1^ region are attributed to C–O–C stretching vibrations, further confirming the ester functionality of the polymer.^42^ The broad peak near 1450 cm^–1^ arises from C–H bending vibrations of methyl and methylene groups present in EGDA and PCLA segments.^43^ Furthermore, a broad absorption band extending over 2800–3400 cm^–1^ is evident, which is assigned to O–H stretching vibrations. These O–H peaks originate mainly from adsorbed moisture and indicate water uptake by the samples from ambient humidity.^43^ No distinct absorption bands were detected in the low wavenumber range (∼750–950 cm^–1^) in the spectrum of the reference sample (R0). On the other hand, by the incorporation of BaTiO_3_ into the polymer matrix, it was clearly observed that new bands appeared in this region and the intensity of existing bands increased with the BaTiO_3_ concentration. These emerging bands originate from the Ti–O and Ba–O lattice vibrations specific to the perovskite structure of BaTiO_3_.^44,45^ The absorption bands observed particularly in the 200–600 cm^–1^ range have been associated with the stretching and bending vibration modes of the octahedral TiO_6_ units in the BaTiO_3_ crystal lattice.^46,47^ A significant increase in the intensities of these vibrational bands was observed with increasing BaTiO_3_ ratio in the polymer matrix, indicating that BaTiO_3_ was successfully incorporated within the polymer matrix, and a direct correlation exists between the loading ratio and the band intensities. Furthermore, the small shifts and peak broadening observed in these bands can be interpreted as the result of possible physical interactions between these ceramic particles and the polymer chains (e.g., hydrogen bonds or interfacial adsorption) or microstresses occurring at the particle–matrix boundary regions.^45^

### 4.9 Mechanical Properties

Uniaxial tensile and three-point flexural tests were performed on SLA-printed specimens (Figure 9) of the neat resin (R0) and BaTiO_3_-filled composites (B0.5, B1.0, B1.5). Each formulation was tested in triplicate (*n* = 3); results are reported as mean ± SD.

**Figure 9:**
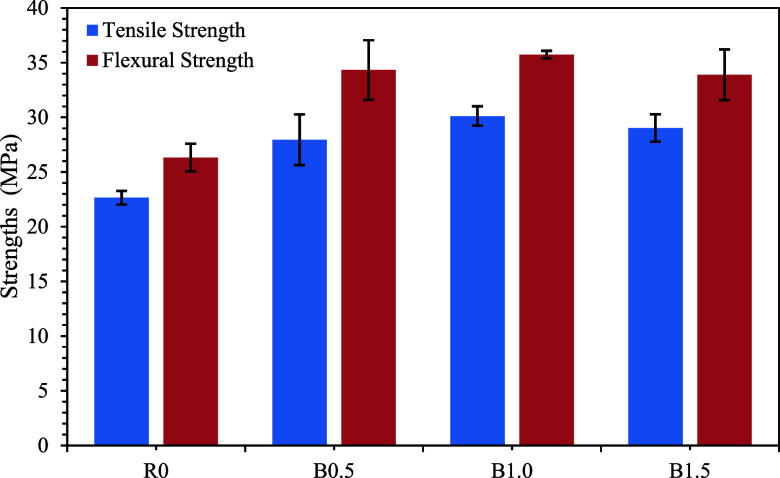
Tensile and flexural strengths of the printed samples with different BaTiO_3_ contents with error bars (mean value ±standard deviation).

The neat resin reached ≈22.7 MPa (R0). Incorporating BaTiO_3_ increased the strength to ≈28.0 MPa at 0.5 wt % (B0.5) and to a maximum of ≈30.1 MPa at 1.0 wt % (B1.0), followed by a slight decrease to ≈29.0 MPa at 1.5 wt % (B1.5) (Figure 9). The improvement up to 1.0 wt % is consistent with efficient load transfer from the matrix to rigid ceramic nanoparticles and enhanced constraint of the polymer network.^48^ The small decline at 1.5 wt % is plausibly linked to partial agglomeration and increased resin opacity/viscosity, which can limit UV penetration and cause locally under-cured regions acting as stress concentrators.^39^ The relatively stable tensile behavior in the R0 group can be attributed to homogeneous UV curing and the absence of foreign particles. On the other hand, the improvements in the BaTiO_3_-loaded groups are directly associated with the reinforcing effect of rigid ceramic nanoparticles, which improve load transfer within the polymer matrix.^48^

Flexural data exhibit the same composition-dependence, with higher absolute values than in tension (Figure 9). Strength rise from ≈26.3 MPa (R0) to ≈34.3 MPa (B0.5) and ≈35.7 MPa (B1.0), then modestly decrease to ≈33.9 MPa (B1.5). The consistently larger flexural than tensile strengths are expected for polymers and their composites, because three-point bending engages primarily the outer fibers (tension/compression) and failure initiates within a smaller stressed volume than in uniform tension. In our series, the flexural-to-tensile ratio is ∼1.16 for R0 (26.3/22.7) and remains >1 for all filled systems, in line with general polymer mechanics.

Considering both loading modes, 1.0 wt % BaTiO_3_ (B1.0) provides the best balance of reinforcement and printability/curing, delivering ∼20–30% gains relative to R0. Beyond this level, marginal decreases are observed, consistent with the common nanocomposite scenario in which the benefits of rigid, well-dispersed nanoparticles are offset at higher loadings by particle clustering, rising viscosity, and increased light scattering during photopolymerization.^48^

### 4.10 Dielectric & Loss Tangent Properties

The dielectric constant (ε_r_) is a key material property that quantifies the ability of a substance to store electrical energy in the presence of an external electric field. It is defined relative to the permittivity of vacuum (ε_r_ = 1) and reflects the material’s polarizability. A higher ε_r_ value indicates enhanced ability to store charge due to increased polarization.

In this study, dielectric measurements were performed on three specimens from each group. However, for the B0.5 group, one replicate was damaged during transport; thus, the reported value represents the average of two measurements. The dielectric constants measured across various frequencies for samples R0, B0.5, B1.0, and B1.5 are summarized in Figure 10.

**Figure 10:**
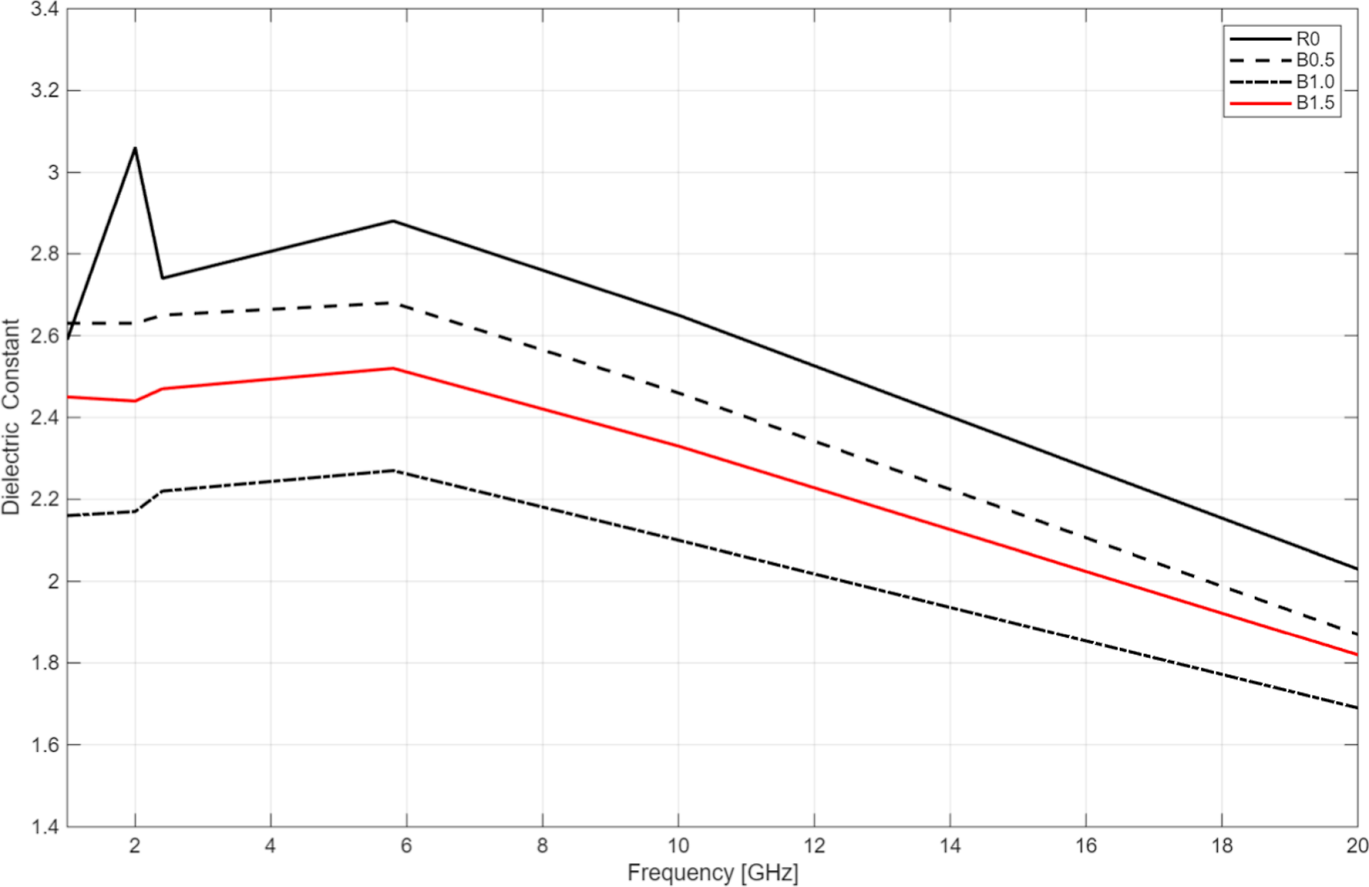
Dielectric constant values of the printed samples with different BaTiO_3_ contents. For loss tangent (tan δ) analysis, all groups were tested in triplicate, and the results are listed in Figure 11. The loss tangent indicates dielectric losses in the material; a lower tan δ signifies better energy efficiency.

As seen in Figure 10, the dielectric constant of all samples decreases with increasing frequency—particularly beyond 5.8 GHz. Notably, among the BaTiO_3_-loaded samples, B0.5 exhibits the highest ε_r_ across all tested frequencies. Despite this, the pure resin (R0) consistently shows a higher ε_r_ compared to its nanoparticle-reinforced counterparts. This unexpected behavior suggests that BaTiO_3_ incorporation, under the current processing conditions, does not significantly enhance the dielectric storage capability of the matrix.

Although BaTiO_3_ typically increases ε′, in our composites the ceramic fraction is well below the percolation threshold required for significant permittivity enhancement. Slight particle agglomeration and increased UV scattering at higher BaTiO_3_ contents further limit interfacial polarization. Consequently, the effective ε′ decreases despite the intrinsically high permittivity of BaTiO_3_. This trend, though counterintuitive, is consistent with reports on dilute BaTiO_3_/polymer systems.^49^

As shown in Figure 11, tan δ increases with frequency, especially beyond 2.4 GHz. Interestingly, all BaTiO_3_-containing groups (B0.5, B1.0, B1.5) demonstrate lower loss tangent values than R0 at certain frequencies, pointing to improved energy retention in those regimes.

**Figure 11:**
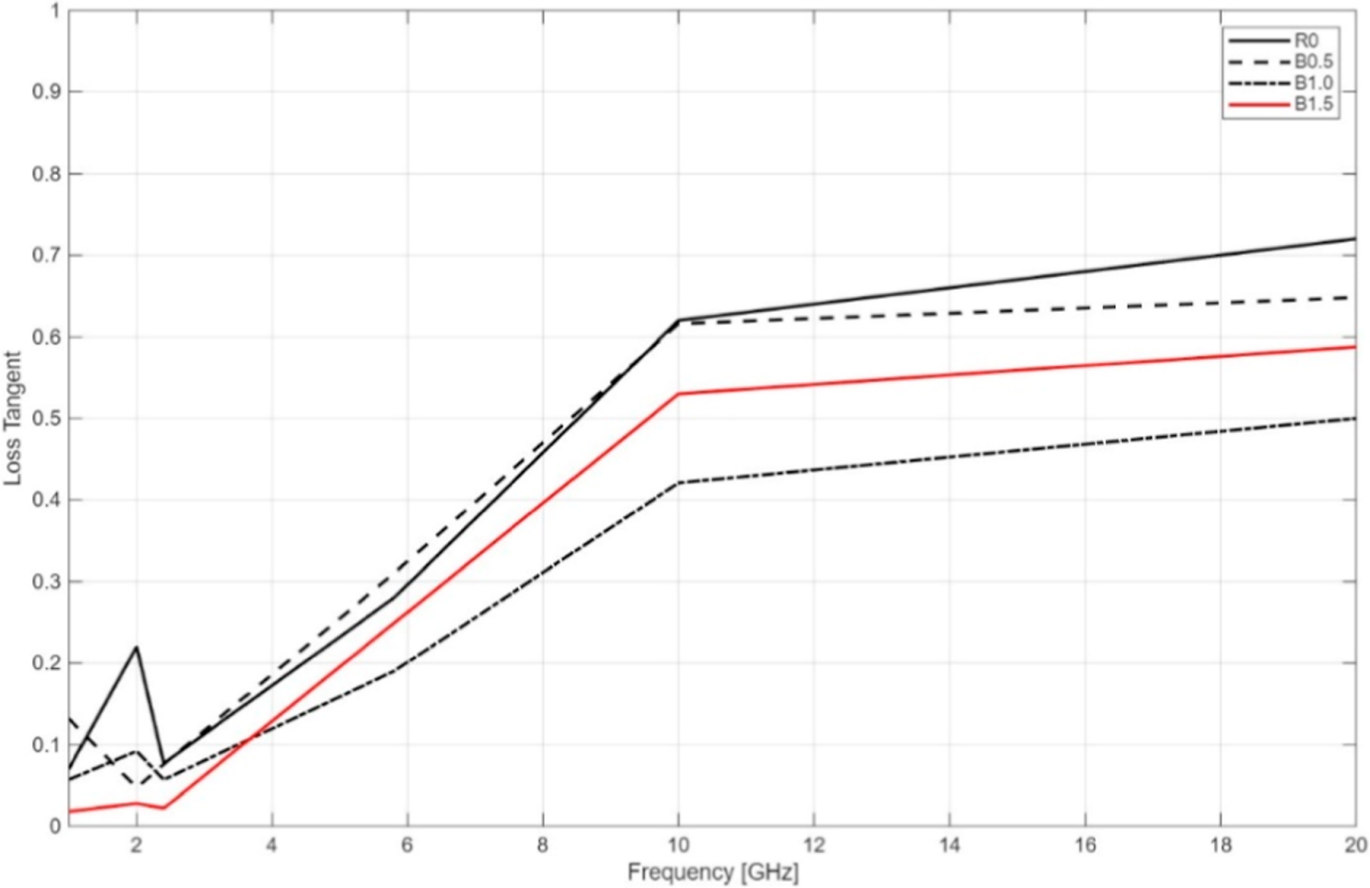
Loss tangent (tan δ) values of the printed samples with different BaTiO_3_ contents.

At 2 GHz, the B0.5 sample exhibits the lowest tan δ (∼0.047), making it suitable for low-to-mid frequency applications. However, a rapid increase in tan δ is observed at 10 and 20 GHz, indicating significant energy dissipation at higher frequencies. The B1.5 group shows the lowest loss tangent in the 1–2.4 GHz range, suggesting superior dielectric efficiency and minimal power loss in this band. The B1.0 sample demonstrates a balanced dielectric profile, with moderate ε_r_ and tan δ values. This group maintains good dielectric performance up to 5.8 GHz, making it well-suited for medium-frequency applications.

When compared with relevant studies in the literature, the dielectric and loss tangent properties observed in this study reflect both the benefits and limitations of incorporating BaTiO_3_ nanoparticles into a photopolymer matrix without surface modification. Chen et al.^50^ reported a high dielectric constant of ∼9.5 and low loss tangent (∼0.02) at 1 kHz in a PEGDA-based BaTiO_3_ nanocomposite fabricated via DLP. However, these values are specific to low-frequency conditions, and direct comparison with the present study’s 1–20 GHz measurements must consider the well-established frequency dependence of ε_r_ and tan δ. Similarly, Wang et al.^51^ achieved ε_r_ values exceeding 10 in SLA-printed composites at 1 MHz using up to 20 vol % BaTiO_3_, yet no surface modification was applied, leading to dispersion-related mechanical limitations. Xiang et al.^52^ by applying silane surface modification to BaTiO_3_ microparticles in an epoxy matrix, significantly enhanced dielectric performance—reaching ε_r_ ≈ 30 and reducing tan δ—highlighting the critical role of interfacial engineering. In contrast, the present study showed that while moderate dielectric values were obtained (e.g., ε_r_ = 2.68 at 5.8 GHz for B0.5), the lack of surface treatment and possible agglomeration hindered further enhancement, especially at high frequencies. Additionally, the pure resin (R0) exhibited surprisingly high ε_r_ across all frequencies, underscoring the potential of matrix-related polarization in high-frequency regimes. Overall, the comparative analysis reveals that the dielectric performance of polymer-based nanocomposites is influenced not only by the intrinsic properties of the fillers and matrix, but also by key microstructural and processing-related factors, including: (i) filler dispersion homogeneity,^53^ (ii) matrix–filler interfacial compatibility,^54^ (iii) nanoparticle size, shape, and surface chemistry,^55^ (iv) filler volume fraction,^55^ and (v) operating frequency band.^56^

Despite using tetragonal-phase BaTiO_3_ nanoparticles, known for their high ε_r_ at low frequencies, the overall enhancement in dielectric constant was limited. Possible explanations include: (i) nanoparticle agglomeration and nonuniform distribution,^57^ (ii) insufficient interfacial bonding due to lack of surface modification^58^ and, (iii) high-frequency test conditions (1–20 GHz), where BaTiO_3_’s permittivity advantage diminishes.^57^

These findings emphasize the importance of interface engineering and microstructural optimization to fully exploit the dielectric potential of ceramic nanoparticles in photopolymer matrices for high-frequency applications.

## 5 Conclusion

In this study, a series of SLA-printed biocompatible nanocomposites reinforced with barium titanate (BaTiO_3_) nanoparticles were successfully developed and characterized for potential application in high-frequency medical electronics. The research employed a comprehensive two-stage experimental design—initially optimizing stereolithography (SLA) printing parameters using pure bioresin, followed by the incorporation of BaTiO_3_ at varying concentrations (0.5, 1.0, and 1.5 wt %) to evaluate their influence on mechanical, thermal, morphological, and dielectric properties.

The findings demonstrated that incorporating BaTiO_3_ nanoparticles into the SLA-compatible bioresin significantly enhanced the mechanical performance, with the 1.0 wt % BaTiO_3_ nanocomposite (B1.0) exhibiting the highest tensile strength (∼30.1 MPa) and flexural strength (∼35.7 MPa). Scanning electron microscopy (SEM) and EDS analysis confirmed that this optimal concentration also provided the most homogeneous particle dispersion, indicating improved matrix–filler interaction. From a dielectric perspective, although BaTiO_3_ is widely recognized for its high intrinsic permittivity and was expected to increase the overall ε_r_ of the composites, the experimental results revealed a counterintuitive trend: the addition of BaTiO_3_ led to a slight reduction in ε_r_ across all tested frequencies compared to the neat resin. This behavior was attributed to the low filler fraction, minor nanoparticle agglomeration, and UV light scattering during printing (especially at ≥ 1.0 wt % BaTiO_3_), which together limited the composite’s effective polarization. Importantly, however, a significant improvement was observed in the dielectric loss tangent. In the 1.0–5.8 GHz range, the BaTiO_3_-loaded samples (particularly B1.0 and B1.5) showed markedly lower tan δ than the pure resin. The 1.0 wt % sample B1.0 achieved the most favorable dielectric profile, reducing energy dissipation while maintaining an acceptable dielectric constant. In practical terms, this balance of properties indicates that the BaTiO_3_-reinforced nanocomposite can meet the demands of GHz-frequency medical sensor applications by providing sufficiently high permittivity (≈2–3) along with low losses.

Among all compositions, the 1.0 wt % BaTiO_3_ nanocomposite (B1.0) exhibited the best overall performance: it struck an optimal balance between mechanical strength enhancement and dielectric loss reduction without sacrificing processability. B1.0s moderate dielectric constant (∼2.2 at 2.4 GHz, vs 2.7 for neat resin) coupled with ∼70% lower tan δ makes it a strong candidate for next-generation biosensors, and telemetry devices in the GHz range. Thermal analyses (TG-DTG) and FTIR spectroscopy further confirmed the structural integrity and stability of the nanocomposites—for example, BaTiO_3_ inclusion led to slightly improved thermal stability (with a higher decomposition temperature and residual mass corresponding to the filler content), although all samples still underwent >90% total weight loss due to the polymer-dominated composition. Overall, this work highlights that a 1.0 wt % BaTiO_3_ addition is optimal for achieving the most effective balance between printability, mechanical robustness, and dielectric performance in SLA-fabricated bionanocomposites.

The presented nanocomposite system—leveraging additive manufacturing precision and functional ceramic reinforcement—offers a promising platform for the development of next-generation biosensors, and high-frequency diagnostic devices. Future studies may focus on advanced surface functionalization strategies, exploring high-aspect-ratio BaTiO_3_ fillers (e.g., nanosheets or nanowires) to enable higher effective loadings, in situ monitoring of particle dispersion, and expanded dielectric testing across wider frequency bands to unlock the full potential of these multifunctional materials in biomedical engineering.

## Supplementary Material



## Notes

Supporting Information includes: Table S1. Comparative mechanical and dielectric properties of the printed samples for pure bioresin samples (R0–R7) (PDF)
