# Supplementary material for: SLA-Printed BaTiO3–Reinforced Bio-Nanocomposites: Influence of Printing Parameters on Mechanical, Dielectric, and Thermal Properties
Source: ACS Omega. 2025 Oct 30;10(44):53350–63. doi: 10.1021/acsomega.5c08292 (PMC12612948; doi:10.1021/acsomega.5c08292)
Supplement: Supplementary file 1 [file ao5c08292_si_001.pdf]

SLA-Printed $BaTiO_3$-Reinforced Bio-Nanocomposites: Influence of Printing Parameters on Mechanical, Dielectric, and Thermal Properties

Tarlan Mahouti[1,*], Ece Örnek[2], Beste Çevir[2], Hale Berber[1], Mehmet Ali Belen[3], Hasan Sadıkoğlu[2], Hakan Yılmazer[1,4,*]

[1] *Yildiz Technical University, Department of Metallurgical and Materials Engineering, 34220, Istanbul, Turkiye*

[2] *Yildiz Technical University, , Department of Chemical Engineering, 34220, Istanbul, Turkiye*

[3] *İskenderun Technical University, Faculty of Engineering and Natural Sciences, Department of Electrical and Electronics Engineering, 31200, Hatay, Turkiye*

[4] *Health Biotechnology Joint Research and Application Center of Excellence, 34220, Istanbul, Turkiye*

**Corresponding Authors: T.Mahouti: tarlan.mahouti@gmail.com*

H.Yilmazer: hakanyil@yildiz.edu.tr.com, yilmazerh@gmail.com

Table S1: Mechanical and Dielectric Test Results for Pure Bio-Resin Samples (R0–R7)

**Sample**	**R0**	**R1**	**R2**	**R3**	**R4**	**R5**	**R6**	**R7**
**ε′) @ 1 GHz**	2.59	2.42	2.28	2.24	2.62	2.08	1.85	2.15
**ε′) @ 2 GHz**	3.06	2.57	2.39	2.40	2.76	2.20	1.98	2.25
**ε′) @ 2.4 GHz**	2.74	2.57	2.40	2.40	2.76	2.22	1.99	2.26
**ε′) @ 5.8 GHz**	2.88	2.69	2.48	2.49	2.83	2.35	2.11	2.39
**ε′) @ 10 GHz**	2.65	2.47	2.30	2.29	2.61	2.17	1.95	2.20
**ε′) @ 20 GHz**	2.03	1.92	1.80	1.80	1.98	1.71	1.58	1.73
**tan δ @ 1 GHz**	0.07	0.07	0.02	0.05	0.05	0.08	0.04	0.07
**tan δ @ 2 GHz**	0.22	0.23	0.15	0.19	0.24	0.26	0.24	0.28
**tan δ @ 2.4 GHz**	0.077	0.08	0.05	0.04	0.08	0.10	0.09	0.11
**tan δ @ 5.8 GHz**	0.28	0.20	0.23	0.25	0.31	0.23	0.18	0.23
**tan δ @ 10 GHz**	0.62	0.56	0.51	0.51	0.63	0.47	0.39	0.48
**tan δ @ 20 GHz**	0.72	0.65	0.58	0.58	0.72	0.55	0.46	0.57
**Tensile Strength Avg (MPa)**	22.65	–	10.91	11.29	–	10.70	11.12	10.20
**Flexural Strength Avg (MPa)**	26.33	–	16.28	18.93	–	–	–	–
